# HLA Typing from 1000 Genomes Whole Genome and Whole Exome Illumina Data

**DOI:** 10.1371/journal.pone.0078410

**Published:** 2013-11-06

**Authors:** Endre Major, Krisztina Rigó, Tim Hague, Attila Bérces, Szilveszter Juhos

**Affiliations:** Omixon Biocomputing, Budapest, Hungary; Seoul National University College of Medicine, Korea, Republic of

## Abstract

Specific HLA genotypes are known to be linked to either resistance or susceptibility to certain diseases or sensitivity to certain drugs. In addition, high accuracy HLA typing is crucial for organ and bone marrow transplantation. The most widespread high resolution HLA typing method used to date is Sanger sequencing based typing (SBT), and next generation sequencing (NGS) based HLA typing is just starting to be adopted as a higher throughput, lower cost alternative. By HLA typing the HapMap subset of the public 1000 Genomes paired Illumina data, we demonstrate that HLA-A, B and C typing is possible from exome sequencing samples with higher than 90% accuracy. The older 1000 Genomes whole genome sequencing read sets are less reliable and generally unsuitable for the purpose of HLA typing. We also propose using coverage % (the extent of exons covered) as a quality check (QC) measure to increase reliability.

## Introduction

One of the driving forces of large-scale sequencing studies has been hunting for mutations that can be associated with genetic diseases [1]. In particular, among the mutation patterns found by these genome-wide association studies (GWAS), different genotypes of the human leukocyte antigen (HLA) genes are showing significant correlation to resistance or susceptibility to particular conditions like AIDS [2], type 1 diabetes [3] or other diseases [4]. The HLA proteins are part of the immune system and play a role in response to infection, some diverse monogenic disorders, auto-immunity, cancer, transplantation, and adverse drug response. The HLA region represents the genome's highest concentration of potential biomarkers for most studied diseases. Specific HLA genotypes have already been associated with sensitivities to five marketed drugs and are currently being investigated as biomarkers in several clinical trials [4].

Using next generation sequencing (NGS) for HLA typing has lagged behind due to some challenges specific to this region. HLA genes are the most polymorphic part of the human genome. There are several thousand known alleles, and most individuals are heterozygous in most HLA loci. In addition, this region contains segmental duplications which are significantly longer than the read-length and insert sizes achievable with current sequencing technologies, and there are a number of similar pseudogenes. Finally, the genetics for some loci are complex (heterodimeric proteins with multiple possible genes). This all contributes to making the classic reference-based alignment of NGS reads unreliable.

The first attempts to determine the HLA type from NGS data used relatively long Roche-454 reads from targeted sequencing data [5]–[7]. Illumina data with shorter read lengths have less frequently been used for HLA typing [8]–[10] and Illumina reads obtained from other studies like whole-genome or whole-exome studies have rarely been used for this purpose. Large-scale sequencing efforts, such as the 1000 Genomes (1KG) project [11] are fundamentally aimed at population genetics and are not intended to precisely genotype individuals. However, as the 1KG project shares some samples with the HapMap project [12] and in some of the previous studies HapMap samples were used for the HLA typing experiments, reference values for HLA typing are available for some of the samples in the 1KG project. This made it possible to compare HLA types obtained using previously developed and validated methods and the corresponding types calculated from the public 1KG paired Illumina short-read data.

In this paper we present results of a feasibility study for HLA typing from whole genome and whole exome paired Illumina data, concentrating on MHC class I genes HLA-A, HLA-B and HLA-C. Although we attempted to determine the type of this three genes from every sample, it became apparent that in many cases there are simply not enough reads to get results for all the loci. Therefore, our other purpose is to determine the current limitations of both the experimental and computational aspects of this procedure, and provide simple measures for quality control for maximal accuracy. To achieve our goals we have selected short read sets from the public 1000 Genomes data repository with known HLA types from the HapMap survey and developed an algorithm to align reads to the IMGT/HLA [13] database and interpreted the results to get the most likely allele set for individuals. We also discuss the limitations of this method and provide directions for further improvements.

## Results and Discussion

### Concordance to samples measured by SSO

Our most important results are that - for experiments passing the QC filters (see section "Quality check measures for correct typing" below) - more than 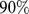 concordance for whole-exome samples, and nearly 80% concordance for low-coverage whole-genome samples could be achieved (Table 1) based on a comparison with known 4 digit typing results [5], [12]. Spreadsheets with calculated and validation values are in Table S1 for whole-exome and whole-genome datasets. Erlich et al. [5] conducted a similar validation for HapMap samples using 454 technology, and even corrected some types that were originally determined by sequence specific oligonucleotide (SSO) hybridization. The HLA types of HapMap samples in this corrected allele list that overlap with HapMap samples sequenced by the 1KG project were used as a reference in our HLA typing experiments. There is still a chance that some of our mistypings are due to mistakes in this corrected reference table, but according to our knowledge these are the most accurate validation values available up to this date.

**Table 1:** Concordance values for samples passing both quality check values.

Samples passing QC	Whole exome		Whole genome	
	Nr. of alleles typed	Concordance%	Nr. of alleles typed	Concordance%
HLA-A	340	92.3	52	78.9
HLA-B	360	96.7	60	85.0
HLA-C	322	92.6	24	95.8
All the genes	1022	93.9	136	84.6

Quality check (QC) was applied in these subsequent steps: i) only those samples were processed where the length of the read was longer than 76 bps for both ends ii) after alignment the percentage of the covered region (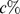) was calculated for exons 2 and 3. If any of these exons were covered by reads only less than 70% of the extent of the exon, the sample failed QC and was discarded. iii) finally, using this formerly calculated 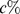 value, if the average 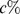 for exons 2 and 3 was less than 80%, the sample was discarded. Average coverage depth was not considered since 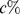 was a better indicator of correct concordance.

All our typings have at least 4-digits, and 6-digit precision is available for most samples. Since no validation data was available for the 6-digit HLA types, we did not investigate the results at this depth of resolution. Typing with 8-digit resolution was not attempted, as most whole genome sequencing runs had insufficient coverage even on the exons of the studied HLA genes and due to the nature of the sequencing project, intron data was obviously not available for the whole exome runs. Still, the results are promising, especially because the experiments were not HLA-targeted but produced by generic large-scale sequencing runs.

### Quality check measures for correct typing

**Figure 1:**
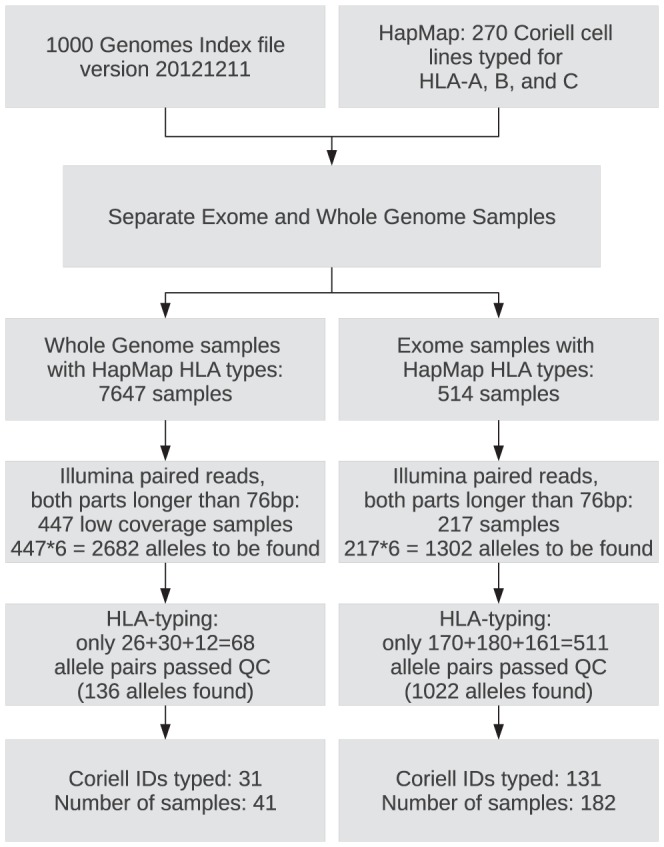
Filtering work-flow. There 1000 Genomes index file was first filtered for paired Illumina samples. There are 270 Coriell cell lines in the HapMap set, from the 1000 Genomes samples we had to select only those IDs which are among these cell lines. After separating the whole genome and whole exome sequencing experiments, these two types of samples were analyzed separately since the average coverage depth is very different for the two datasets. Those samples where the readlength was less than 76 base pairs for any part of the pair were thrown away and was not processed further. Finally, HLA typing was successful only for samples that were passing the coverage QC measures. The first such measure was that the coverage % for either exons 2 or 3 had to be at least 70% – if any of the exons was covered in less extent, the typing for that gene (for both alleles) was discarded. Furthermore, typing also failed if the average coverage % calculated for exons 2 and 3 was less than 80%.

The index file of the 1KG experiments (version 20121211) was filtered for datasets containing reads of paired Illumina sequencing and Coriell IDs of HapMap samples in the reference allele list. After typing the filtered sets, it became apparent that some experiments are not suitable for our purposes due to short readlength and/or low coverage. Therefore, we had to establish general quality check (QC) measures which can be used for each sample in order to achieve reliable HLA typing. There are 270 Coriell IDs in the HapMap database, but at the end of QC check there were only 31 Coriell IDs (41 samples) for whole-genome experiments and 131 Coriell IDs (182 samples) for whole-exome experiments left – many Coriell cell lines were sequenced more than once. Figure 1 explains the details of this filtering process.

**Figure 2:**
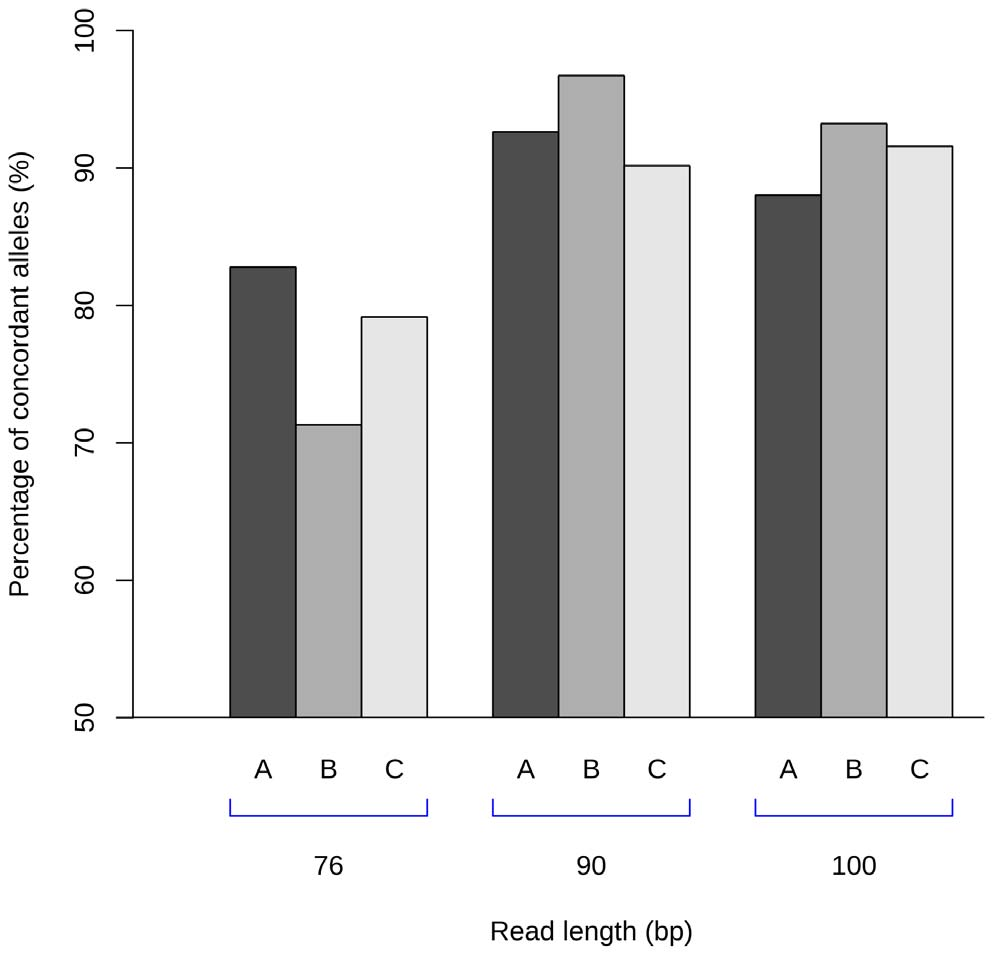
Typing concordance versus read length. Read length can serve as the very first quality check measure; pairs with having reads shorter than 76 bps are showing very high ambiguity and mistyping. Even 76 bps is a lower bound for correct typing, samples with shorter read lengths are less concordant. Picture showing concordance and read length for all the 217 whole exome samples; there were 360 typings (HLA-A,B,C both alleles) with readlength 76 bps, 366 typings for samples with readlength 90 bps and 514 typings for 100 or 101 bps reads (one of the HLA-C typing for 100 bps samples was unsuccessful giving no typings at all.)

The very first quality check measure was based on read length; only experiments where the reads were longer than 76 bases for both reads in each pairs were used. Having shorter reads resulted in ambiguous alignments, and NGS technology producing shorter reads than this can be regarded as obsolete. Most of the samples have reads of length 90 or 101 bps (Table 2). Read length shows clear correlation to concordance: this can be seen on Figure 2 depicting only whole-exome samples. Better concordance for longer reads is not surprising, low typing accuracy for 76 bps reads indicates that from practical point of view at least 76 bps reads or even 100 bps or longer reads are needed to have reliable results. Correlation coefficients between concordance values and read length were calculated using the Kendall rank correlation 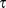 method [14]. For HLA-A, the correlation coefficient didn't significantly differ from zero (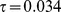, 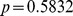). For HLA-B and HLA-C, the correlation tests were significant (HLA-B: 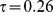, 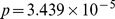, HLA-C: 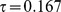, 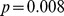). The correlation test was also significant for per sample concordance (i.e. the summarized concordance for all HLA-A, B and C alleles of a sample; 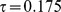, 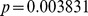).

**Table 2:** Read length distribution for different experiments.

Sequencing	76bps	90bps	100–108 bps	Total
Whole genome	302	8	137	447
Whole exome	60	61	96	217

Read length was the first crucial quality check value. Only paired samples were considered having reads longer than 76 bps on both part of the pair. Reads shorter than 76 bps were practically useless: most of the mistyped samples had shorter read lengths.

**Figure 3:**
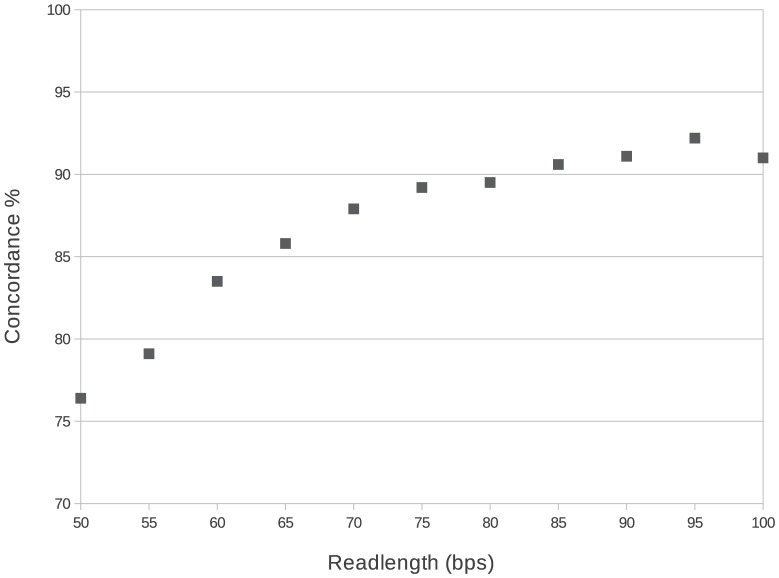
Effect of read length for typing concordance. Using samples having 100 bps long read we simulated a set of read by trimming reads to shorter lengths. Typing a series of reads with different readlengths shows that concordance rises with longer readlength, and 90% concordance can be achieved using 75bps long reads. Therefore, samples with shorter read pairs were not used in our experiments.

Since the reads in the 1000 Genomes samples have either 76, 90 or 100 bps length, using 182 samples with 100 bps readlength we simulated a series of pairs with different readlength and calculated the concordance for this subset. The 100 bps long reads were trimmed to 50, 55, 60, 65,... basepairs lengths and these trimmed sets were typed as real samples. The result of this simulation was in accordance with the previous observation: readlength is a strong determinant of concordance, reads shorter than 75 bps will give lower than 90% concordance and longer readlength will give only a slight increase. E.g. the concordance gain between 50 bps and 60 bps long reads is 7.1%, between 60 bps and 70 bps long reads is 4.4% but a mere 1.4% between 75 bps and 85 bps reads (Fig. 3).

For NGS one of the most frequently used measures is the depth of coverage (number of reads covering a particular base position). For exons of the relatively short and highly variable HLA genes this measure can be misleading: even if the number of reads covering the gene is high on average, there can be a small important region distinguishing alleles that is not covered by any reads. The most polymorphic parts of genes HLA-A, B and C are the second and third exons, proper coverage for these exons is crucial for correct typing. Therefore, as our next QC measure we have introduced coverage percent(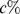), which is the extent of the exon covered by reads calculated as the percentage of the length of the exon. These 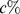 values were used in two steps; the first criteria for passing the QC was that, for each gene both exons 2 and 3 had to be covered at least 70% of their length, or, in other words the 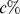 needed to be higher than 70% for both exons 2 and 3. In ideal case these two important exons are covered by reads in their whole lengths. Whole exome and whole genome samples are not always fulfilling this, therefore, we had to make a compromise and introduce a threshold 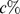 value that results in acceptable concordance values.

**Figure 4:**
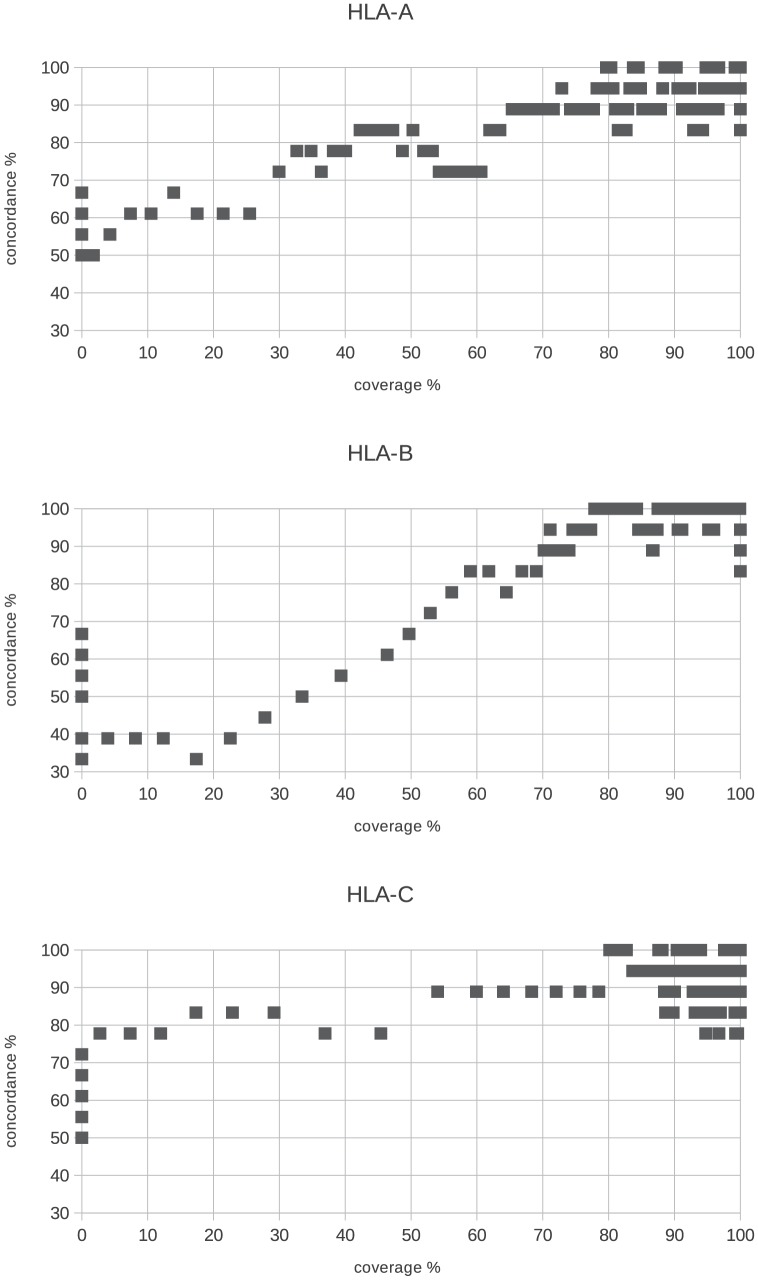
Minimal coverage % for exons 2 and 3. To pass the first 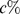 QC filter we expected for every sample that the 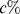 for both exons 2 and 3 were higher than 70%. It was because when plotting concordance vs. 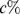 the concordance is higher than 90% when the 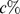 is at least 70% and there is no strong improvement using higher values.

This minimal 70% threshold is suitable because when inspecting the relationship between minimal coverage % and concordance for all the three genes, we can see that around this threshold the concordance saturates and reaches 90%. Higher minimal 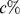 values do not necessarily gives better concordance for all the genes (see Figure 4). If this condition was not fulfilled, both allele calls for the given gene were discarded due to failed QC.

**Figure 5:**
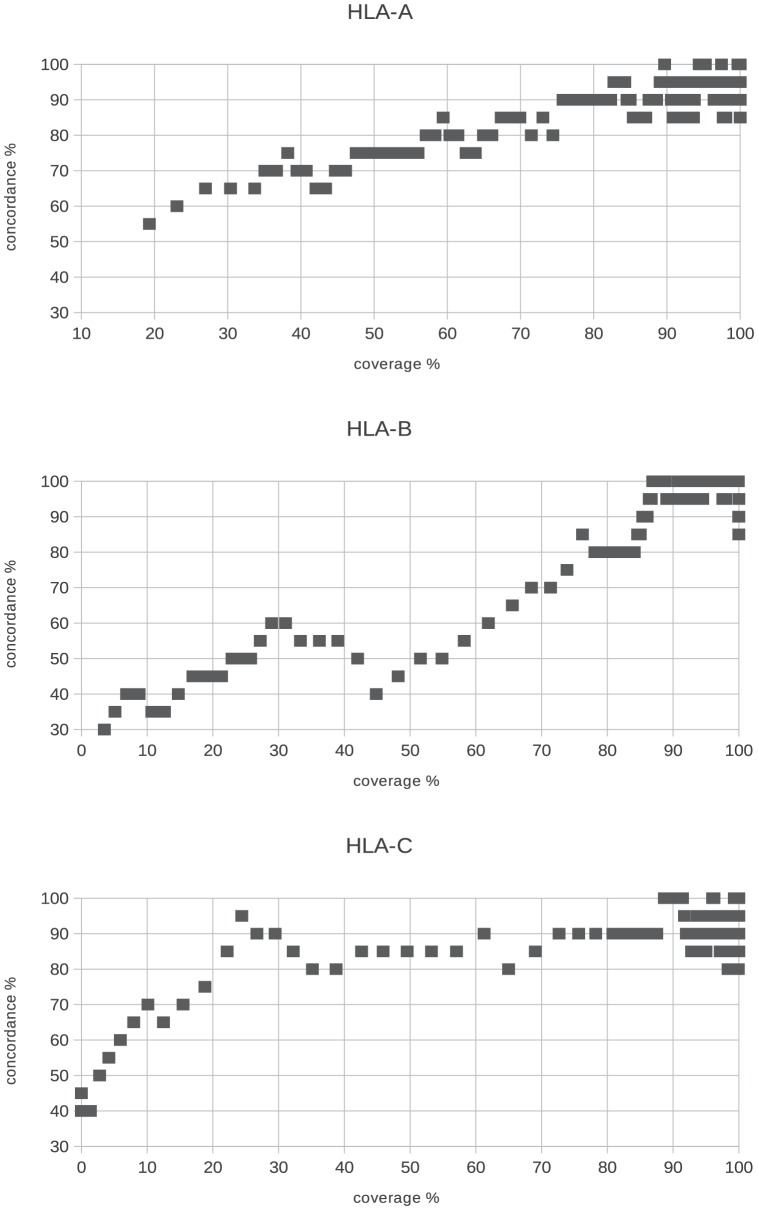
Minimal average coverage % for exons 2 and 3. For the second QC filter based on 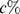 we expected that the average 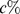 for exons 2 and 3 has to reach 80%. The concordance is around 90% for this 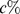 value and higher concordance could be reached only if the sample size decreases significantly.

The next criteria we used as a quality check filter was that - in order pass QC - 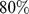 of exons 2 and 3 had to be covered on average. For example 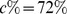 for exon 2 and 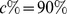 for exon 3 would pass, but 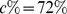 and 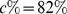 respectively for exons 2 and 3 would fail. Using this average 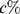 threshold we intended to have an acceptable coverage % for both important exons. Figure 5 is similar to the previous graph showing that at 80 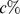 the concordance approaches or reaches 90%. Higher average 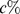 threshold would give better concordance (especially for HLA-B) but we would have lower sample number. It must be emphasized that for clinical samples (i.e. for bone marrow transplantation) it is expected to have always 100% coverage for both of these exons.

Coverage depth was generally low for whole-genome samples and a magnitude higher for whole-exome samples. This was one of the reasons to prefer coverage % as a QC filter: although coverage depth is indeed an important measure, it is not sufficient by itself to adequately filter out the low-concordance candidates. According to our results, an average coverage depth of only 3 reads could be a good QC candidate, as on average whole-genome samples having this coverage depth has more than 80% concordance. However, almost all the samples failing to have this depth of coverage are also failing on the other QC measures. Those few samples having low coverage depth but passing the coverage % QC filters were not discordant, but many samples having higher coverage depth than 3 and failing on the coverage % filters were discordant. We have concluded that it is advised to use only the coverage % as a QC measure.

These simple QC filters are rather valuable tools to find out the limits of the typing algorithm. Most of the whole-genome samples were discarded due to these filters; on the other hand, the majority of the whole-exome samples passed the QC filters. In practice, to have highly concordant typing, 95% or higher coverage % on all the exons 2 and 3 of HLA-A, B and C genes is recommended. Obviously, exons other than 2 and 3 also have significance in typing but according to our experience the coverage on these two exons is the most important limiting factor in our work-flow.

### Mistypings - issues of common and rare alleles

**Figure 6:**
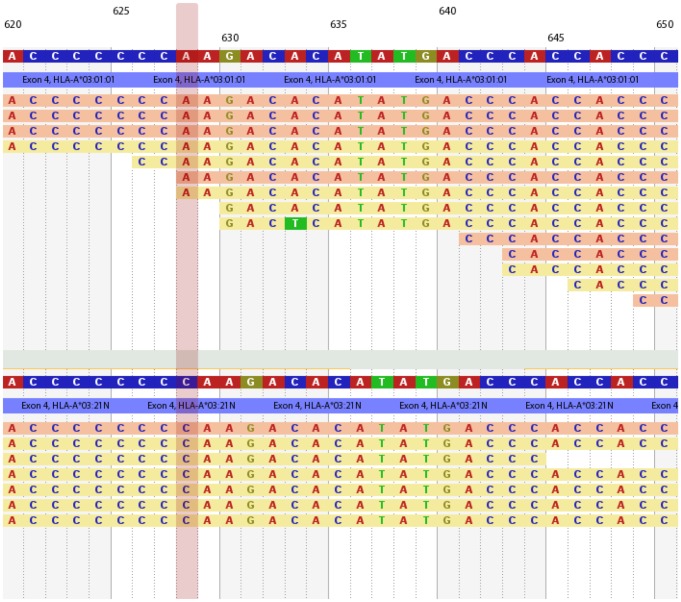
Difference in HLA-A*03:01:01:01 and HLA-A*03:21N alignments. Both alleles show relatively good coverage, but reads covering HLA-A*03:21N exon 4 (the distinguishing part between the two alleles) are from other genes and pseudogenes like HLA-H, or HLA-B,C and E. Mistyping is mostly due to this phenomenon when analyzing whole-exome or whole-genome samples; reads from other regions are brought in as "alignment noise". This in most cases result in mistyping to a rare allele, though in some unfortunate cases to a different common one. Mistyping tends to be systematic: valid types are usually mistyped to the same rare allele.

Exome samples provided wider stretches of exons covered and deeper coverage, therefore, we have investigated mistypings only for these samples. In 32 cases of the total 62 mistypings the mis-types reported were rare alleles and some of these have been assigned rather systematically to certain common types (Table 3). According to the common and well-documented HLA alleles catalogue [15] there is only a single allele in our whole validation set that can be considered rare (HLA-B*41:04 for samples with Coriell ID NA19223). Furthermore, our rare discordant types are representing only 8 alleles in total. For example we systematically mistyped HLA-A*03:01 to HLA-A*03:21N in several samples. The latter null allele is indeed a rare one, and their sequences differ only in a single cysteine (C) insert in a homopolymer region at the start of exon 4 of the allele; HLA-A*03:01 has seven Cs and HLA-A*03:21N has eight Cs there. Looking at the alignment pattern it is clear that there are quite a few reads matching exactly to both of these alleles either with seven or eight C nucleotides. Homopolymer errors are unlikely for Illumina sequencing but are still present [16], therefore, we decided to check this by simulating reads using Stampy [17], not allowing indels and using read qualities from the actual problematic data sets. The reference for simulation was the HG19 MHC region (chr6:29,677,000-33,486,000) representing the PGF haplotype, having HLA-A*03:01:01:01 [18]. When typing this simulated data by our algorithm, we expect to report identical HLA-A*03:01:01:01 alleles, however, we reported two alleles HLA-A*03:01:01:01 and HLA-A*03:21N. The first hundred bases of HLA-A*03:21N on exon 4 are very similar to the corresponding region of the HLA-H pseudogene and also match to certain parts of HLA-B, C and E. Therefore, these reads are mapping to many locations, polluting the statistics and bringing mistypings into final results (see Figure 6).

**Table 3:** Mistyping of different alleles.

	Correct allele	Mistyped allele	Number of cases	Mistyped allele frequency
1	HLA-A*01:01	HLA-A*01:11N	3	rare
2	HLA-A*02:06	HLA-A*02:01	4	common
3	HLA-A*03:01	HLA-A*03:21N	8	rare
4	HLA-A*11:01	HLA-A*11:50Q	4	rare
5	HLA-C*07:01	HLA-C*07:18	5	common
6	HLA-C*08:01	HLA-C*08:22	10	rare
7	HLA-C*18:01	HLA-C*18:02	4	common

Usually a common allele is mistyped as a rare allele, and the wrong type is systematically the same rare allele. These mistypings are due to reads from other parts of the genome, pseudogenes or other HLA genes having similar sequences. However, there are cases when common alleles are mistyped as other common ones: mistyping is systematic in these cases also.

An other example of systematic mistyping is HLA-C*08:01 to HLA-C*08:22, the latter being a rare allele with few reported occurrences world-wide to this date [19]. These two alleles differ only in a single SNP on exon 6, the exonic sequence for 08:22 is gtggaaaaggagggagctActctcaggctgcgt and for 08:01:01 is gtggaaaaggagggagctGctctcaggctgcgt (uppercase letters emphasizing the SNP difference). A sequence search either in ENSEMBL [20] or UCSC [21] reveals that the sequence that corresponds to the common allele is rather unique. On the other hand, this part of the rare allele can be mapped to quite a few places in the genome, mostly to genes and pseudogenes like HLA-B, HLA-F, HLA-J or HLA-H. Bearing in mind that our data comes from whole exome or whole genome sequencing we concluded that again, reads from other, similar parts of the genome are brought in during typing.

This phenomenon unfortunately affects common alleles as well; the most frequent mistyping involving two common alleles were HLA-C*07:01 to HLA-C*07:18, HLA-A*02:06 to HLA-A*02:01 and HLA-C*18:01 to HLA-C*18:02. In fact, HLA-C*07:01 was mistyped to two other common alleles, to HLA-C*07:19 and HLA-C*07:26 as well. For the HLA-C*07:01 to HLA-C*07:18 mistyping case our software identified the correct allele as a ''runner up'' in the candidate list with 6 digits precision as HLA-C*07:01:01. The coding sequences of HLA-C*07:01:01 and HLA-C*07:18 again differs only in a single SNP on exon 6 of HLA-C. A thorough search of this sequence (gtggaaaaggagggagctgctctcaggYtgcgt) shows that it is indeed not only present in HLA-E, but also in a non-MHC gene called DNTT, located on chromosome 10. The scenario is similar to the case when HLA-A*02:06 is mistyped as HLA-A*02:01; although there are two SNPs that discriminate the two alleles on exon 2, very similar sequences can be found in HLA-B, C, G, H, and HLA-L. This shows that sometime the algorithm prefers another alleles, either rare or common, because reads from other parts of the genome or exome are brought in during the alignment. However, it is relatively easy to spot these discrepancies. As the concordance is around 90% even with the current approach, we decided to tackle this problem in the next release of the software.

An other possible source of mistyping is that in our present approach we are using only the coding sequence parts of the allele references. Although many genomic references including introns are available in the IMGT/HLA database, there are still quite a few sequences with only the exonic parts – for some only exons 2 and 3 are defined. To achieve high accuracy (correct typing) and precision (get six or eight digit typings) the intronic part should be included on the long term. Dealing with missing exons and introns needs a more complex *de novo* approach that is beyond the scope of the current study and is under active development [8].

Our results support the original assumption: using paired Illumina reads from whole genome or whole exome experiments it seems to be possible to determine the MHC-I HLA types with around 90% or higher concordance. Typing is likely not possible for all types of experiments though; appropriate coverage depth is needed and, based on our findings, it seems to be even more important to cover the whole extent of exons 2 and 3 of HLA-A, B and C in order to have high concordance. Having many validated examples from the HapMap project, we have been able to establish quality check measures to indicate possible mistypings. By introducing two simple coverage % measures, we achieved higher than 80% concordance for low-coverage whole genome samples and higher than 90% percent concordance for whole exome sequencing. Of course, as other data sets can have significantly different characteristics than the HapMap samples used in this validation project, the coverage measures have to be refined for each set of data. Our findings show that HLA typing can be performed even without specific HLA primers, although the results from whole genome or whole exome experiments will likely not be as accurate and precise as needed for clinical application. It must be kept in mind that some mistypings will likely occur: most of these are usually due to data from pseudogenes and repeats that are present in non-HLA-targeted sequencing experiments. Result of these whole genome and exome typings can be used for population studies i.e. for disease and drug response association studies involving HLA types. To make the typing more accurate, in the forthcoming versions of the software we are planning to address the aforementioned systematic mistypings, introduce intronic sequences into typing and generally increase concordance by testing new sequencing technologies with longer reads.

## Materials and Methods

Samples for whole-genome and whole-exome sequencing were treated differently during the analysis because the sequencing strategy, coverage and read length were so different for these experiments that their results and overall accuracy was significantly divergent.

One of our original goals while developing our HLA-typing method was to provide software that can be run on a commodity desktop computer using only moderate resources i.e. can be used with limited disk space, memory and CPU power. All the raw sequencing data from the circa 650 paired samples obviously will not fit into the disk drive of a PC designed for daily desktop use. Therefore, we have pre-filtered all the sets to leave only those reads that are mappable to the IMGT/HLA database. The filtering software selected those pairs where at least one of the reads can be aligned with no more than three mismatches and one soft-clip to at least one allele in the database and the orientation of the mapped reads is forward-reverse if both reads in the pair is aligned. Pairs that can be mapped only with indels were also discarded. Since the alignment part of our algorithm is exactly the same for both filtering and typing, we have used these filtered sets for further analysis. The number of pairs filtered out for the exome sets was between 10K to 50K, meanwhile for the whole-genome experiments the yield after filtering was about ten times less (i.e. only a few hundreds of pairs). The size of the filtered whole-exome dataset is around 1.9Gb (uncompressed) which is easily manageable. Due to their low coverage, whole genome samples produced much less data, the size of the filtered datasets is a mere 80 Mb. These smaller, filtered whole-genome and whole-exome read sets are available for download from https://s3.amazonaws.com/omixon-publication/hapmap_hla/HapMap_1KG_HLA_suppl_filtered_reads.tgz (about 700 Mb compressed).

Our search algorithm is conceptually similar to those from some previous studies [5], [9] but instead of BLAST and/or gapped Smith-Waterman alignment we are looking for reads that are able to align to any of the sequences in the IMGT/HLA database (version 3.10 10/2012) [13] with no or very few mismatches (allowing for soft clips at read ends). Any read that could not be aligned to an allele without indels, or contained too many mismatches, was discarded. The number of allowed mismatches was derived from the read length:
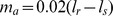



where 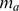 is the number of mismatches allowed, 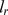 is the readlength, 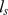 is the length of the soft-clip (if any). Reads were only aligned to the exons in the IMGT/HLA reference allele sequences, this allowed us to achieve up to six digits resolution. After alignment, for each reference allele's coding sequences the coverage depth and coverage % values were sorted. In the next step we filtered allele candidates using all this allele coverage data, and left only those candidates that have high enough number of reads covering the allele. This filter discards putative alleles having too few reads covering the reference and/or having long parts or whole exons not covered at all. Finally, having narrowed down to only few dozens of possible individual alleles, we are searching for allele pairs in a way that we are optimizing for both coverage depth and coverage %, reporting allele pairs that contain both a high number of mapped reads and have adequate coverage of exons for both alleles at each locus. There is a chance that we have more than one set of pairs containing the same number of reads and coverage pattern (for example alleles differing only in intronic regions); in this case we are reporting these as ambiguous candidates.

## Supporting Information

Table S1 **Calculated and measured HLA-A, HLA-B and HLA-C types for HapMap samples.**
(XLS)
